# Metabolomics: Perspectives on Clinical Employment in Autism Spectrum Disorder

**DOI:** 10.3390/ijms241713404

**Published:** 2023-08-29

**Authors:** Martina Siracusano, Lucrezia Arturi, Assia Riccioni, Antonio Noto, Michele Mussap, Luigi Mazzone

**Affiliations:** 1 Department of Biomedicine and Prevention, University of Rome Tor Vergata, Via Montpellier 1, 00133 Rome, Italy; 2 Child Neurology and Psychiatry Unit, Department of Neurosciences, Policlinico Tor Vergata Hospital, Viale Oxford 81, 00133 Rome, Italy; lucrezia.arturi@gmail.com (L.A.); assiariccioni@gmail.com (A.R.); luigi.mazzone@uniroma2.it (L.M.); 3 Department of Biomedical Sciences, University of Cagliari, Cittadella Universitaria, SS 554, Km 4.5, 09042 Monserrato, Italy; 4 Department of Surgical Sciences, School of Medicine, University of Cagliari, Cittadella Universitaria, SS 554, Km 4.5, 09042 Monserrato, Italy; 5 Systems Medicine Department, University of Rome Tor Vergata, Montpellier Street 1, 00133 Rome, Italy

**Keywords:** autism spectrum disorder, metabolomics, biomarkers, clinical profile, phenotype, sleep disorders, gastrointestinal problems, food selectivity, early detection, metabolism

## Abstract

Precision medicine is imminent, and metabolomics is one of the main actors on stage. We summarize and discuss the current literature on the clinical application of metabolomic techniques as a possible tool to improve early diagnosis of autism spectrum disorder (ASD), to define clinical phenotypes and to identify co-occurring medical conditions. A review of the current literature was carried out after PubMed, Medline and Google Scholar were consulted. A total of 37 articles published in the period 2010–2022 was included. Selected studies involve as a whole 2079 individuals diagnosed with ASD (1625 males, 394 females; mean age of 10, 9 years), 51 with other psychiatric comorbidities (developmental delays), 182 at-risk individuals (siblings, those with genetic conditions) and 1530 healthy controls (TD). Metabolomics, reflecting the interplay between genetics and environment, represents an innovative and promising technique to approach ASD. The metabotype may mirror the clinical heterogeneity of an autistic condition; several metabolites can be expressions of dysregulated metabolic pathways thus liable of leading to clinical profiles. However, the employment of metabolomic analyses in clinical practice is far from being introduced, which means there is a need for further studies for the full transition of metabolomics from clinical research to clinical diagnostic routine.

## 1. Introduction

Autism spectrum disorder (ASD) is a neurodevelopmental disorder characterized by childhood-onset impairment in social communication, associated with restricted and stereotyped behaviors [1].

The phenotype heterogeneity of this neurodevelopmental disorder arises at different levels: clinical (severity of core symptoms and associated behaviors) and medical (co-occurring conditions including, above all, sleep disorders and gastrointestinal disorders). This heterogeneity is reproduced at the molecular level in a mirrored way; that is, to each clinical or medical phenotype corresponds a specific molecular phenotype.

From a clinical point of view, the variability in cognitive, adaptive and language abilities observed among individuals with ASD leads to two main clinical phenotypes strictly linked to the level of cognitive, adaptive skills and the level of support required: high (HF-ASD)- or low-functioning (LF-ASD) [2,3]. Moreover, the behavioral profile of an individual with autism ranges from more to less presence of repetitive and restricted (RRBs) patterns of behavior, atypical sensorial responsiveness, cognitive rigidity (including food selectivity) and sociocommunicative impairment [4,5]. The variability in ASD clinical phenotypes contributes to challenges in the diagnostic process and may represent a crucial factor implicated in individual responses to treatment [4].

From a clinical point of view, autism is associated with a consistent number of co-occurring medical and psychiatric conditions. In particular, individuals with ASD are 3.5 times more likely to suffer from gastrointestinal issues (GIs) in comparison with typical-development peers (TD) [6], while the estimated prevalence of sleep disorders is around 13% of the ASD population, in comparison with 3.7% in the general population [7]. Furthermore, co-occurrent conditions of epilepsy, attention deficit and hyperactivity disorder (ADHD), anxiety and mood disorders are frequently observed [8].

To date, the diagnosis of autism is clinically based, albeit supported by the use of standardized observation tools and questionnaires/interviews (e.g., the autism diagnostic observation schedule, second edition—ADOS-2; the autism diagnostic interview-revised—ADI-R). As a matter of fact, specific and reliable biomarkers are not available yet. The identification of biomarkers for ASD may be useful in supporting clinicians in the improvement of the diagnostic process. No single protein, enzyme or metabolite can be considered as a candidate biomarker of autism; rather, a panel of biological constituents might meet criteria for the detection and follow-up of specific autistic phenotypes.

Genetic analyses (chromosomal microarray, fragile X testing) are recommended as a first instrumental examination in case of ASD diagnosis [9]; however, genetic mutations explain only 10–20% of cases of autism, while a portion of cases are due to common polymorphism [10,11].

For this reason, scientific research is moving toward the identification of specific autism biomarkers—immune, hormonal and neurophysiological—and, even if promising results have been proved, to date there are no established markers. 

Serotonin [12,13], oxytocin [14] and immune system dysregulation are some of the pieces of evidence that emerged from biofluid-based analyses, which, however, lack disease specificity. 

**Figure 1:**
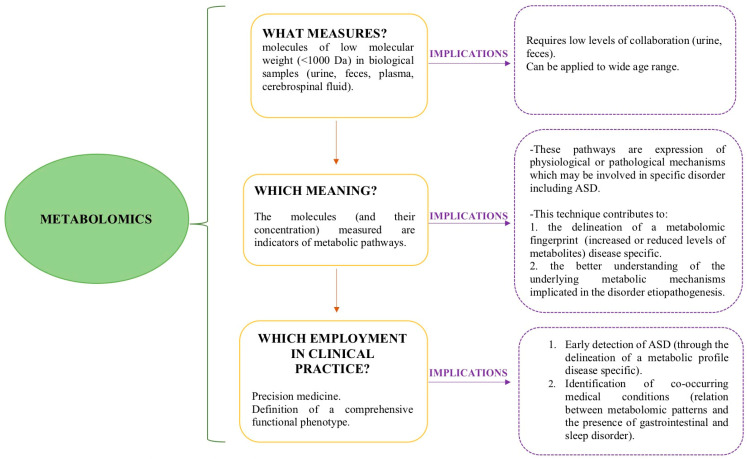
A brief illustrated description of metabolomics and its clinical implications.

Since there is a need to establish markers for ASD, *-omic sciences* (genomics, epigenomics, transcriptomics, proteomics, metabolomics), and in particular metabolomics, offer promising perspectives, representing an expanding discipline in current autism research [15]. Metabolomics is a technique which permits the identification and detects the presence of whole sets of molecules of low molecular weight (<1000 Da) in biological samples (urine, feces, plasma, cerebrospinal fluid) [16]. One of its strong points is represented by its ability to snapshot the rapid daily variations in metabolic issues due to clinical, therapeutic and lifestyle changes [15]. Metabolomic tools present the possibility to assess the complex relation between the etiology of a disease and the physiology of an organism [17], providing a comprehensive functional phenotype through the integration of clinical features with both genetic and nongenetic risk factors [18] (Figure 1). Considering metabolomic techniques as based on biological sample collection, their noninvasiveness represents another strong point of this approach, especially in the context of individuals with ASD. Several studies have aimed to detect a metabolic fingerprint of ASD [19,20,21,22,23] and to identify metabolic pathways related to co-occurring medical conditions [24,25].

The available literature on metabolomics and ASD is wide-ranging; however, to the best of our knowledge, it lacks narrative reviews mainly addressed to the clinicians (child psychiatrists, pediatricians and child neurologists) working in the field of ASD (diagnosis, follow-up, medical care and treatment). 

Thus, the present overview aims to summarize and discuss the current literature on the application of metabolomic techniques in the ASD clinical population, focusing on three different levels:

1. Investigate the metabolomic profiles of individuals with ASD in order to improve early diagnosis;
2. Highlight possible relations between metabolomic patterns and symptom severity profiles among individuals with ASD;
3. Identify the association between metabolomic patterns and the presence of co-occurrent medical conditions in the ASD clinical population.

Therefore, this narrative review contributes by giving an interpretation of the employment of metabolomic techniques in clinical practice on autism.

A revision of the current literature regarding the application of metabolomic analysis on individuals with ASD was carried out in order to give an overview of the available data on this topic.

Two authors carried out this research independently, consulting PubMed, Medline and Google Scholar. “Autism”, “neurodevelopment”, “medical comorbidities”, “phenotype”, “metabolomics”, “metabolome profile” and “biofluids” were selected as keywords for the research. The following were considered as inclusion criteria: original works in the English language, studies conducted on human samples (more than 10 individuals), full text available, and a focus on the three aims in the ASD population (metabolomic profile and early ASD diagnosis; metabolomic patterns and symptom severity profiles; and metabolomic patterns and co-occurrent medical conditions). On the other hand, we excluded commentaries, editorials, protocols, papers written in languages other than English and studies conducted on animal models.

Table 1, Table 2 and Table 3 were developed in order to address the main objectives of this study.

Table 1 includes studies that investigate the use of metabolomic analyses for the early identification of ASD (AIM 1).

Table 2 includes studies employing metabolomic analyses to characterize the clinical ASD phenotypes in terms of core symptom severity (AIM 2).

Table 3 includes research investigating the possible use of metabolomics to identify co-occurring medical conditions among the ASD clinical population (AIM 3). 

## 2. Discussion

**Figure 2:**
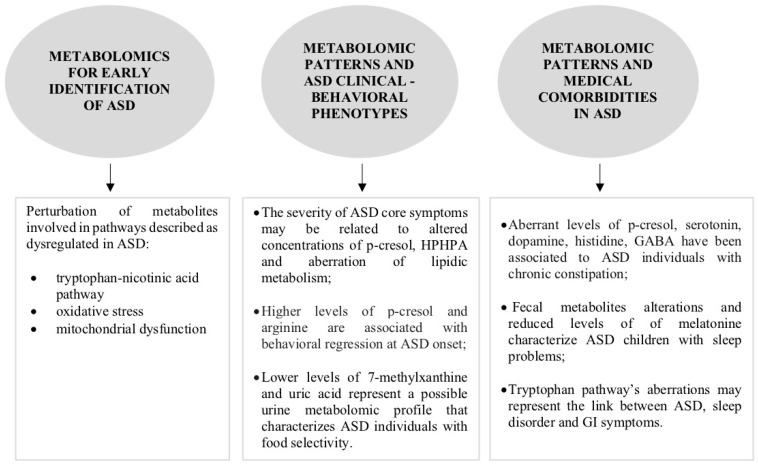
Summary of main findings of the overview.

Within this study, we give an overview of the literature on metabolomics and ASD, analyzing the possible applications of this technique in the context of this neurodevelopmental disorder, specifically in the following contexts: early diagnosis; characterization of the clinical ASD phenotype; and detection of co-occurring medical conditions among the ASD clinical population (Figure 2; Table 4). A total of 37 relevant articles published in the period 2010–2022 was included in the overview. Overall, the selected studies included a total of 2.079 individuals diagnosed with ASD (1.625 males, 394 females; mean age of 10,9 years), 51 individuals with other psychiatric comorbidities (developmental delays), 182 at-risk individuals (siblings, those with genetic conditions) and 1.530 healthy controls (TD). Sixteen of these studies were carried out in Europe [21,22,24,25,29,30,31,35,37,39,41,44,45,47,52,56], twelve in Asia [23,28,33,34,36,40,42,43,46,50,54,55,58], eight in the USA [27,32,38,48,49,51,53,57] and one in Australia [58].

### 2.1. What Is the Role of Metabolomics in the Early Identification of ASD?

Early signs of autism emerge in the first 12 months, even though the average age of ASD diagnosis is still around the 4th year of life [59,60]. Improving earlier diagnosis and starting earlier intervention represent priorities to optimize the outcome; however, the identification of ASD signs in toddlers remains a challenging issue for clinicians, because some children, especially those with HF-ASD, may not show clinical manifestations at earlier ages [61].

In this context, the application of metabolomic analyses may represent a promising technique for the early diagnosis of ASD (in case of identification of a specific metabolic marker of autism), especially in at-risk populations—defined as conditions characterized by an increased possibility of being affected by autism, such as familiarity with ASD (especially for siblings of autistic individuals), genetic syndromes (such as fragile X syndrome and tuberous sclerosis complex) and maternal disease (e.g., maternal immune activation) [62,63].

The metabolomic technique, measuring the level of specific metabolites in biofluids, indirectly evaluates the pathways’ functioning in which the metabolites are involved. Research shows that the metabolic pathways most commonly described as altered in individuals with ASD are as follows: amino acid and nicotinic acid metabolism, mitochondrial dysfunctions and antioxidant status, in addition to the role played by gut microbiota-related metabolites [64]. Furthermore, other alterations frequently observed in the ASD clinical population include aromatic amino acids—precursors of neurotransmitters—and hormone levels implicated in nervous system regulation (catecholamine, dopamine and serotonin were also frequently observed) [15]. Table 1, Table 2 and Table 3 summarize the main metabolomic studies, providing a metabolomic profile for individuals with ASD in comparison with the general population and those with at-risk conditions.

Specifically regarding **amino acid metabolism**, evidence highlights aberrations in glutamate, tyrosine and homocitrulline in the ASD population [23,26,29,32,40,42,43]. However, the results are still inconsistent, probably due to the variability in age, sex, symptom severity and the presence of other co-occurrent conditions in the selected samples and the lack of a properly matched control group [22,24,26,30,31,32,43,64,65].

The tryptophan–nicotinic acid pathway (N-methyl-2pyridone-5-carboxamide, N-methyl nicotinic acid and N-methyl nicotinamide) is typically perturbated in ASD [26]. This pathway is involved in oxidative stress and in sleep/wake cycle regulation (see the next paragraph for implications specifically concerning sleep disorders in ASD). The reduced conversion of tryptophan to melatonin can determine an increase in nicotinic acid synthesis, with a greater susceptibility to oxidative stress [31,64]. Imbalances in urinary L-threonic acid (oxidant) and carnosine and urate (antioxidant metabolites) are expressions of increased oxidative stress [27,31]. It is noteworthy that oxidative stress and mitochondrial dysfunctions [28,66,67] are mechanisms involved in both social and cognitive functioning [68,69], domains generally impaired in ASD.

However, even if several metabolic aberrations—as described above—have been detected within individuals with ASD, some metabolic pathways are shared among disorders (Down syndrome, idiopathic developmental delays) [38], making it difficult to define a specific metabolic profile for ASD.

Finally, if by now the early identification of ASD symptoms (in the first years of life) represents a challenge at both the clinical and molecular levels, an even more intriguing research domain is constituted by the pre-, peri- and postnatal periods [37,44,70].

To date, the application of available technologies for metabolome profiling (e.g., proton nuclear magnetic resonance spectroscopy, liquid chromatography, gas chromatography with mass spectrometry) gives the possibility to explore early metabolome variations when carrying out peri- and postnatal screenings [71]. Recently, a longitudinal newborn screening [44], conducted by analyzing the metabolomic profiles (blood samples collected between 3 and 10 days from birth and later biobanked) of 74 newborns, revealed higher levels of methacholine (a synthetic choline ester that acts as a nonselective muscarinic receptor agonist) in newborns later diagnosed with ASD. However, even if this result is promising, the possible role of this metabolite in the context of ASD still remains largely unknown [72].

Summarizing the evidence concerning the first aim of this overview, most of the studies report alterations in metabolite concentration reflecting perturbed pathways known to be dysregulated in ASD: the tryptophan–nicotinic acid pathway, oxidative stress and mitochondrial dysfunction.

To draw conclusions, we postulate that, nowadays, the role of metabolomics in the early identification of ASD is still under development and its clinical utility remains questionable. As a matter of fact, the identification of a disease-specific metabolomic fingerprint could aid and support the clinician in the diagnostic process—however, not alone but always in association with clinical warning symptoms diagnosis. However, the goal is to identify a metabolite panel associated with the risk of ASD; this objective will enable the possibility to include a metabolomics-based neonatal screening in babies identified as at risk to develop autism.

### 2.2. Can Metabolomics Contribute to the Clinical Phenotype Stratification of ASD?

ASD’s clinical phenotype is characterized by extreme variability in terms of the level of ASD’s core symptoms’ severity (social affect, repetitive and restricted behaviors including sensorial hypo- or hyper-responsivity) and associated behaviors (aggression, anxiety). Phenotype heterogeneity is strictly linked to outcome variability [71,73]. Thus, the identification of a metabolomic fingerprint associated with a distinct clinical profile (phenotype stratification) could contribute to the delineation of clinical trajectories and to the creation of tailored interventions [74]. Table 2 summarizes the main metabolomic studies addressing this topic.

Recently, a positive relationship was reported between the severity of autism symptoms and the urinary levels of the following metabolites: 2-hydroxyacrylic acid and 3-(3-hydroxyphenyl)-3-hydroxypropionic acid (HPHPA), *p*-cresol, benzoic acid, hippuric acid and 3-hydroxypentanoic acid [21]. Interestingly, all discriminant metabolites found in this study were strictly linked to dietary habits, gut dysbiosis and mitochondrial dysfunction. In fact, most of these metabolites (2-hydroxyacrylic acid, HPAPA, 3-hydroxypentanoic acid) are produced within the gastrointestinal tract through the intervention of gut bacteria.

***P*-cresol**—an organic aromatic compound (4-methylphenol) for which exposure commonly occurs through skin contact, inhalation, food and beverages—has raised the interest of researchers as a promising biomarker for ASD. Several studies have demonstrated high levels of *p*-cresol in the urine and feces but not in the blood of autistic individuals [65,75] and a significant correlation between the magnitude of the *p*-cresol increase and the severity of symptoms [47]. Although *p*-cresol is produced by at least 55 phylogenetically divergent bacterial strains belonging to the gut microbiota [76], robust evidence demonstrates a strong association between *p*-cresol excess and *Clostridia* spp. overgrowth in the gastrointestinal tract, especially involving *C. difficile* [47].

The administration of *p*-cresol in mice causes autism-like symptoms strikingly resembling core symptoms and comorbidities in autistic individuals [77]. *P*-cresol and its conjugated derivative *p*-cresylsulfate are toxic compounds inducing multiorgan injuries and dysfunctions; in particular, the kidney, the liver, the endothelium, the immune system and the central nervous system (CNS) are the main targets. The neurotoxicity of *p*-cresol consists of the impairment of dendritic development, synaptogenesis and synapse function in hippocampal neurons, as observed in rat cell cultures [78]; in addition, *p*-cresol causes the irreversible inhibition of the enzyme dopamine-beta-hydroxylase (DBH), a critical enzyme catalyzing the conversion of dopamine to norepinephrine, with a consequent accumulation of dopamine [41,79]. The accumulation of dopamine promotes brain damage due to the excess of unstable dopamine quinones, toxic adducts of dopamine and oxygen superoxide. In turn, these toxicants lead to an increase in oxidative stress and injury to neuronal mitochondria [59].

Altered concentrations of this metabolite have been reported in several studies [53,64,65], showing the role of *p*-cresol as a discriminant metabolite in autism, also in terms of symptom severity profiles. In this context, a *p*-cresol increase was directly associated with ASD’s core symptoms’ severity measured through standardized measures including the autism diagnostic observation schedule-2nd edition [21] and the childhood autism rating scale (CARS) [47]. Interestingly, the authors speculate that, given that a source of *p*-cresol is environmental exposure (paints, disinfectants, solvents, cleaning products), low-functioning individuals could present an excess of this metabolite due to oral exploration common to this clinical profile (they frequently bring objects to their mouth). From this perspective, other studies found a specific association of the metabolite with greater stereotypic and repetitive behaviors [75].

Furthermore, a correlation between clinical profile severity and the fecal metabolome emerged in a recent study on preschooler individuals with autism [24], showing higher levels of amino acids in children with a mild autism profile and higher levels of N-Methylhydantoin, 1,3-Dihydroxyacetone and fucose in children with greater clinical impairment. Finally, amino acid metabolism, monoamine neurotransmitters, γ-Aminobutyric acid (GABA), ornithine and choline have recently been related to core symptoms and aberrant behavior in ASD [48,49,50,51]. In particular, the negative correlation between the GABA (decreased level of inhibitory neurotransmitters) and autism’s core symptoms [41,48,49] supports the theory of an excitatory–inhibitory imbalance in ASD [40,80].

Another crucial metabolite is **3-(3-hydroxyphenyl)-3-hydroxypropionic acid (HPHPA)**, an organic acid deriving from nutritional sources and arising from bacterial metabolism in the gastrointestinal tract, especially from the action of the anaerobic bacteria *Clostridia* spp. HPHPA was found to be elevated in the urine of autistic individuals [81].

Shaw [82] postulated that *Clostridia* spp. producing HPHPA and *p*-cresol could also be involved in the alterations in dopamine and norepinephrine metabolism, promoting oxidative stress and brain damage. HPHPA originates from the bacterial pathway of m-tyrosine, a tyrosine analog promoting autistic core symptoms in animal models. Chronic excess of HPHPA turns this metabolite into neurotoxin and metabotoxin; it is unclear whether the toxic effects are due to the property to mime catecholamines, altering the neurotransmission, or to the capacity to act as an analog to tyrosine and phenylalanine. Our group found that urine HPHPA levels positively correlate with the severity of ASD’s core symptoms, assessed with the ADOS-2 calibrated severity score [21].

***Benzoic acid*** is an organic compound containing a benzene ring that bears at least one carboxyl group. Benzoic acid is a byproduct of bacterial phenylalanine metabolism and is also produced by the bacterial fermentation of polyphenols derived from fruits or beverages taken with the diet. Elevated urinary levels of benzoic acid may indicate a shortage of glycine, the amino acid necessary for its transformation into a hippuric acid by the deamination of the bacterial enzyme phenylalanine. Abnormalities in hippuric acid excretion were found very early in individuals with autism [46], indicating alterations in gut microbiota; in particular, *Clostridia* spp. overgrowth was observed [26,83,84].

The clinical heterogeneity of ASD can be observed from the onset of the neurodevelopmental disorder. Among the ways early ASD symptoms may arise, there is regression—meant as a loss of skills in development, language and/or behavior [85]. To date, little is known about the underlying mechanism of this phenomenon, and this remains one of the most challenging issues in the context of autism. (How frequent is the phenomenon? When does it happen? Why do some children show regression and others do not? How can regression be measured?)

Recently, Rangel-Huerta and colleagues [22] attempted to detect, in the first two years of life, a metabolomic profile related to a clinical condition of regression (identified through a five-item questionnaire based on the ADI-R). Mild differences in the blood levels of endogenous metabolites (including arginine) and lipid metabolism (mono- and diacylglycerols) emerged among the two groups (children with and without regression)**.** Specifically, children with ASD with regression exhibited metabolic profiles characterized by higher concentrations of arginine [22]. Higher levels of this metabolite are thought to be related to the increase in oxidative stress through the nitric oxide (NO) pathway [22,64]. In addition, a recent study [51] (Brister et al., 2022) found that neurodevelopmental regression was associated with changes in nicotinamide and energy metabolism.

Concerning **lipid metabolism**, an accumulation of mono- and diacylglycerols and a decrease in long- and medium-chain fatty acids were found in children with regression. These metabolomic aberrations reflect alterations in triacylglycerol and phospholipid metabolism, fatty acids and β-oxidation usually observed in the ASD clinical population [86,87].

Finally, even the well-known *p*-cresol has been investigated from the perspective of detecting a possible metabolomic fingerprint of regression [47].

To conclude, we have reported the main metabolic aberrations associated with specific clinical profiles: the severity of ASD’s core symptoms and aberrant behaviors (*p*-cresol, HPHPA, amino derivates, lipid and energy metabolism) and behavioral regression at the disorder’s onset (arginine, *p*-cresol, nicotinamide).

However, the key point is as follows: how can a metabolite be a measure of clinical heterogeneity and clinical severity? This question remains mostly unanswered; nevertheless, some speculations may be produced. We have mentioned the main metabolites somehow involved in ASD; however, we should opt for a different perspective, not narrowed and limited to a single constituent, but broadened to the interaction between elements: the individual genotype and multiple environmental factors (i.e., diet habits, gut microbiome). The metabotype may represent such interplay and, in these terms, specific metabotypes—a panel of biological constituents, instead of single proteins, enzymes or metabolites—shared by individuals with ASD may be a better expression of autism’s clinical phenotype in terms of both heterogeneity and symptom severity [88].

Notoriously, within the ASD population, nutrition and food selectivity (meant as a restricted food intake due to sensorial issues including consistency, color, temperature and taste) constitutes a common and challenging issue for the clinical phenotype of individuals with ASD, contributing to clinical heterogeneity and severity. As a matter of fact, food selectivity is tightly linked to environmental factors such as diet habits (the variety and amount of food intake) and intestinal dysbiosis which, in turn, have several implications for the metabotype.

It is worth noting that nutrition and food habits play a key role in the interplay between the microbiome, gut and brain (the so-called gut–brain axis). Food selectivity may influence the gut microbiome; therefore, such an issue reflects the possible link between nutrition, the microbiome, the metabolomic profile and behavior. On one hand, food selectivity induces inadequate nutrient intake (influencing the gut microbiome); on the other, rigidity and repetitive food habits may be responsible for excessive intake of specific food (i.e., carbohydrates), hence again impacting gut microbial composition. Recently, Mussap et al. [21] investigated a possible urinary metabolic profile related to food habits, finding 7-methylxanthine and uric acid as promising metabolites characterizing individuals with ASD and food selectivity.

Such premises suggest that metabolomics can provide an additional contribution to stratifying the ASD population.

### 2.3. Can Metabolomic Patterns Be Indicators of Co-Occurrent Medical Conditions in ASD?

Comorbidities are very frequent among individuals with ASD [89], especially sleep disorders (13%) and gastrointestinal symptoms (GI) (46–84%), suggesting possible shared pathophysiologic mechanisms between autism and these comorbidities [7,10]. Unfortunately, language and communication impairment—characteristic of individuals with autism—represents a strong barrier for the prompt recognition of these medical symptoms. Thus, the identification of specific disease-related biomarkers could help in clinical management.

#### 2.3.1. Gastrointestinal Disorders

A growing body of literature reports a correlation between the presence of GI disorders (e.g., abdominal pain, constipation, chronic diarrhea and reflux) and the behavioral profile (irritability, externalizing problems, behavioral dysregulation and repetitive patterns of behaviors) [90,91].

The relationship between GI symptoms and behavior is debated and represents a key clinical research topic within neurodevelopmental disorders, especially ASD. Certainly, a discomfort that cannot be explained due to communicative difficulties (language and/or cognitive impairment) can lead to behavioral worsening (aggressive behavior, irritability, tantrums, greater repetitive behavior) that are hard to clearly and adequately interpret by families and clinicians. However, there is an established neurobiological link between intestinal microbiota and brain function (the gut–brain axis), mainly explained through the following mechanisms: the direct modulation of the enteric nervous system by gut metabolites; and the production of bioactive metabolites—including neurotoxins and neurotransmitters (i.e., GABA)—induced by the gut microbiota [92].

GI disorders are associated with gut dysbiosis in the general population [93] and also in ASD [94]. Therefore, gut microbiota-derived metabolites—measured through metabolomics—may be indicators of underlying GI disorders within ASD.

Among the most frequent urinary and fecal metabolomic alterations described in the ASD population, there are abnormal levels of hippuric acid, p-hydroxyphenylacetic acid and 3-(3-hydroxyphenyl)-3-hydroxypropanoic acid, propionic acid, *p*-cresol, isopropanol and GABA. These metabolic perturbations may be caused by gut dysbiosis, especially by *Clostridium*, *Alistipes*, *Akkermansia*, *Caloramator* and *Sarcina* spp. overgrowth and the depletion of *Prevotella* spp., *E. siraeum* and *Bifidobacterium* spp. [53,95].

Chronic constipation often characterizes ASD in children and represents a common cause of emergency visits and inpatient admissions [96]. Even concerning this medical condition, *p*-cresol plays a pivotal role. In fact, higher levels of this metabolite have been associated with chronic constipation through two main mechanisms: the increased production of *p*-cresol, mainly due to gut dysbiosis [65]; and the slow intestinal transit time, leading to major absorption of *p*-cresol and resulting in enhanced renal excretion [47]. Both mechanisms are always associated with leaky gut, the most important factor promoting the transit of gut microbial metabolites and toxins from the gut lumen to circulation.

Further metabolite alterations (i.e., organic acid, sugars, hippurate, glycine, tryptophan, creatinine, taurine) have been related to GI disorders within autism [30,35,95].

Recently, Dan and colleagues [55] examined the relationship between the metabolomic profile and microbiota in fecal samples of individuals with ASD affected by chronic constipation. The authors found a significant association between changes in serotonin, dopamine, histidine and GABA concentrations and the depletion of *Sutterella*, *Prevotella* and *Bacteroides* species. Interestingly, given the involvement of the aforementioned metabolites in behavior, the authors claimed that their findings provide a clue for a better understanding of the pathophysiological mechanisms related to autistic behaviors.

Noteworthy, other authors highlight the need to employ with caution metabolomic results (the perturbated concentration of fecal or urinary gut-derived metabolites) as indirect indicators of gut dysbiosis (microflora species richness and diversity)—and subsequently as biomarkers as GI disorders—suggesting a combined use of microbiota analysis and metabolomics [56].

Finally, up to the present, metabolomics (associated or not with microbiomics) does not represent the clinical practice for the detection of GI disorders within ASD and it is not likely to replace the routine instrumental investigations used for the detection of GI conditions (i.e., stool examination, gastroscopy). However, metabolomics may have significant implications for better deciphering the pathophysiological mechanisms underlying the gut–brain axis in autism.

#### 2.3.2. Sleep Disorders

Sleep disorders (e.g., awakenings, parasomnias) have negative impacts on the clinical phenotype of the individual and on the quality of life of the whole family [7,54,97]. A better understanding of the underlying metabolic mechanisms implicated in sleep disorders may be helpful to delineate targeted interventions.

Serotonin and melatonin—and the respective pathways they are involved in—are two key metabolites of the sleep/wake cycle frequently dysregulated in ASD [14,54,98].

Increased concentration of serotonin and lower levels of melatonin and 3-hydroxybutyric acid were reported in individuals with ASD and sleep disorders [14,41,98]. Indeed, the authors described a positive association of sleep outcome measures and the abundance of two key gut bacteria (*Faecalibacterium* and *Agathobacter*), and a negative correlation of sleep disorders with fecal metabolite changes (3-hydroxybutyric acid). This reflects the impact of microbial and metabolic alterations on sleep.

Moreover, alterations in urinary metabolites involved in the tryptophan pathway (decreases in kynurenine and kynurenic acid; increases in quinolinic acid and xanthurenic acid) and subsequently in oxydoreduction processes are frequently reported within ASD [21,25,45], underlying the role of these two mechanisms in autism.

Together, these results, even if not yet consistent, suggest possible shared pathophysiological mechanisms between ASD and sleep disorders and GI disturbances.

## 3. Conclusions

This overview highlights that the use of metabolomic analyses for early detection of ASD symptoms to define a specific clinical profile and to identify co-occurring medical conditions is promising but not yet applicable to the daily clinical diagnostic practice with a person with ASD. Specific metabolic profiles in the serum or urine of autistic individuals have not yet been definitively identified; nonetheless, several metabolic alterations—linked to pathophysiological mechanisms of neurodevelopmental disorders—seem to characterize ASD and co-occurring conditions.

As a matter of fact, even if metabolomics actually does not represent an ASD-specific diagnostic tool, such a technique can already be employed to stratify the ASD population according to distinct clinical profiles (i.e., gastrointestinal disorders, sleep problems, food selectivity).

Several limits affect the currently available literature; they do not permit us to draw reliable conclusions that are relevant for all ASD clinical populations. First of all, the age of autistic individuals is heterogeneous among studies; thus, there is the need to conduct studies with a great number of individuals with ASD divided on the basis of their age (preschoolers, schoolers, adolescents, adults). In addition, studies are not easily comparable due to the utilization of different methods (NMR, GC-MS, LC-MS), approaches (targeted or untargeted), multivariate statistical approaches, biofluids (blood, plasma, urine, feces) and disease severities.

In conclusion, the question of “how can a metabolite be a measure of clinical heterogeneity?” remains mostly unanswered. A plausible response may be that a specific phenotype is not reflected by the single metabolite, but rather by a metabolic profile comprising a set of elements—namely the metabotype. The latter, being possibly indicative of a clinical condition, is by such means a promising approach for ASD.

## Figures and Tables

**Table 1:** This table includes 22 studies that investigate the use of metabolomic analyses for the early identification of ASD (AIM 1).

Early Identification of ASD						
Study	Sample	Aim of the Study	Clinical Tools	Biofluid	Main Metabolomic Findings in ASD	Main Authors’ Conclusions
**Yap et al., 2010 [26]**	39 ASD 28 SIBS 34 TD	To characterize metabolomic profiles of individuals with ASD, unaffected SIBS and TD groups.	-	Urine	↑ **Increased**: acetate, dimethylamine, succinate, taurine, N-methylnicotinamide, N-methylnicotinic acid, N-acetyl glycoprotein fragments, N-methyl-2-pyridone-5-carboxamide ↓ **Decreased**: hippurate, glutamate, phenylacetylglutamine	Children with ASD show alterations in gut microbiota metabolism, amino acid metabolism and nicotinic acid metabolism.
**Ming et al., 2012 [27]**	48 ASD 53 TD	To identify a pattern of metabolic perturbance in ASD.	-	Urine	↑ **Increased**: urocanate, glutaroylcarnitine, 3methylglutaroylcarnitine, 2-(4-hydroxyphenil) propionate, taurocholenate sulphate ↓ **Decreased**: glycine, serine, threonine, β-alanine, histidine, taurine, N-acetylglycine, 3-(3-Hydroxyphenil) propionate, carnosine, urate, 5-aminovalerate	- Increased levels of gut-derived metabolites. - Increased oxidative stress within ASD.
**Kuwabara et al., 2013 [28]**	25 ASD 28 TD	To identify metabolites as potential biomarkers for ASD.	ADI-R GAF Wechsler scales	Blood/ Plasma	↑ arginine, taurine ↓ 5-Oxoproline, lactic acid	Association between deviated plasma metabolite levels, oxidative stress and mitochondrial dysfunction in individuals with ASD.
**Emond et al., 2013 [29]**	26 ASD 24 TD	To identify potential biomarkers of ASD.	-	Urine	↑ **Increased**: citrate, succinate, glycolate ↓ **Decreased**: vanillylhydracrylate, 3-methyladipate, palmitate, stearate, p-hydroxymandelate, 3-hydroxyphenylacetate, phosphate, hippurate, 3-hydroxyhippurate, 1H-indole-3-acetate, 3- methyladipate, 4-Hydroxyphenyl-2-hydroxyacetate	The metabolic fingerprint of autistic children is marked by changes in 15 metabolites, 3 increased and 12 decreased. 1H-indole-3-acetate could be implicated in microbial pathways associated with gut bacterial flora.
**Noto et al., 2014 [30]**	21 ASD 21 SIBS	To classify metabolome alterations between children with ASD and unaffected SIBS.	ADOS-2	Urine	↑ **Increased**: 3-(3-hydroxyphenyl)-3-hydroxypropanoic acid, ribose, 3,4-dihydroxybutyric acid, arabinofuranose, glycolic acid, glycine, cisacotinic acid, phenylalanine, tyrosine, p-hydroxyphenylacetic acid, homovanillic acid, tryptophan, polyols arabitol, xylitol, threitol, pyroglutamic acid ↓ **Decreased**: fructose, 1,2,3-butanetriol, propylene glycol	Elevated urinary concentrations of several sugars and organic acids among children with ASD, probably due to the interactions among diet, genes and microbiome.
**Kałużna-Czaplinska et al., 2014 [31]**	14 ASD 10 TD	To identify alterations in small molecular weight compounds and to find potential biomarkers of ASD.	-	Urine	↑ **Increased**: citrate, L-threonic acid, 2,3-dihydroxybutanedioic acid, α α- hydroxybutyric acid, β-hydroxybutyric acid, m- hydroxybenzoic acid, succinate, oxalic acid, p-hydroxyphenylacetic acid, D-mannitol, ribonic acid, D-arabinitol, L-tyrosine, α-hydroxyglutaric acid, homocisteine ↓ **Decreased**: PPA, phosphoric acid, butanoic acid, L-serine, sebacic acid (acid decanedioic), tryptophan	Discriminant metabolites are known to be involved in multiple biochemical processes, especially in energy and lipid metabolism and oxidative stress. Some of them, such as tartaric acid, are markers of dysbiosis.
**West et al., 2014 [32]**	52 ASD 30 TD	To discover discriminant metabolic features of ASD.	ADOS-G ADI-R SCQ	Blood/ Plasma	↑ homocitrulline, glutamate	Differences in some key metabolites’ abundances (such as homocitrulline) might provide a signature that could distinguish individuals at higher risk for developing ASD.
**Wang et al., 2016 [33]**	73 ASD 63 TD	To identify potential biomarkers for the early diagnosis of ASD.	ABC CARS DQ	Blood	↑ decanoylcarnitine, pregnanetriol ↓ uric acid, docosahexaenoic acid, adrenic acid, sphingosine 1-phosphat	Two metabolites (docosahexaenoic acid and sphingosine 1-phosphat) have potential as biomarkers for the clinical diagnosis and evaluation of ASD.
**Xiong et al., 2016 [34]**	62 ASD 62 TD	To identify potential biomarkers for ASD.		Urine	↑ HPHPA, 3-hydroxyphenylacetic acid, 3-Hydroxyhippuric acid	The measurement of these compounds represents a promising potential clinical tool for ASD diagnosis.
**Lussu et al., 2017 [35]**	21 ASD 21 SIBS	To identify metabolome variations to discriminate between people with ASD and SIBS.	ADOS-G	Urine	↑ glycine, tryptophan, hippurate, creatine, D-threitol ↓ glutamate, taurine, lactate, valine, betaine, cretinine	The urine of children with ASD reflected alterations in neurotransmitters, tryptophan–serotonin pathways, mammalian–microbial cometabolism and oxidative stress metabolism.
**Bitar et al., 2018 [36]**	40 ASD 40 TD	To identify metabolomic disturbances associated with ASD.	CARS	Urine	↑ **Increased**: phosphoserine, glutamic acid, nicotinamide ribotide trigonelline, 5-aminoImidazole-4-carboxamide, riboflavin, glycerol−3-phosphate, cholic acid ↓ **Decreased**: threonine, creatine, serine, N-acetylphenylalanine, tyrosine, hydroxybenzoic acid, hydroxyproline, urocanic acid, cysteic acid, 2-hydroxybutyric acid, citric acid, guanine, N-amidino aspartic acid, acetylcarnitine, methyl acetoacetic acid	Perturbations in tyrosine, 2-hydroxybutyrate, creatine and glutamate, amino acids, carbohydrates and oxidative stress pathways. Perturbations in trigonelline, cysteic acid and guanine.
**Barone et al., 2018 [37]**	83 ASD 79 TD		ADOS-2 ADI-R Standardized battery for IQ/DQ	Blood Urine	↑ citrulline, acetylcarnitine, methylmalonyl/3-OH-isovalerylcarnitine, decanoylcarnitine, tetradecadienoylcarnitine, hexadecanoylcarnitine, octadecenoylcarnitin	Of the 45 analyzed metabolites, 20% were significantly increased in ASD, including the amino acid citrulline and acylcarnitines, indicative of impaired mitochondrial fatty acid β-oxidation.
**Orozco et al., 2019 [38]**	167 ASD 51 DD 31 DS 193 TD	- To investigate the association between metabolic alterations and developmental disabilities. - To understand the specific biological perturbations associated with developmental disorders.	-	Blood	↑ betaine, choline, lactate, cis-aconitate, ornithine, alanine, arginine, asparagine, glycine, histidine, serine ↓ 2-aminobutyrate, 2-hydroxybutyrate, 3-hydroxybutyrate	Despite the varied origins of developmental disabilities, it was observed that there were similar perturbations in one-carbon metabolism (associated with the folic acid–folate cycle) pathways among Down syndrome and ASD cases. Other metabolites and pathways were uniquely associated with DS or ASD.
**Leboucher et al., 2019 [39]**	25 FXS 29 TD	To investigate the systemic consequences of FMRP absence.	-	Blood	↑ free fatty acids ↓ glucose, insulin	FMRP loss increased hepatic protein synthesis and impacted pathways notably linked to lipid metabolism.
**Liu et al., 2019 [40]**	57 ASD 81 TD	To assess the differences in amino acid metabolism between ASD and TD.	-	Urine	↑ arginine, ornithine, 5-hydroxytryptamine, methionine sulfoxide ↓ aspartate, homocysteine, lysine, 5-aminovaleric acid	A possible imbalance between excitatory–inhibitory amino acid metabolism was found in children with ASD.
**Liang et al., 2020 [23]**	22 ASD 22 SIBS	To discriminate the metabolic modifications between ASD and TD.	CARS ABC	Urine	↑ tryptophan, hippurate, glycine, creatine ↓ trigonelline, melatonin, pantothenate, serotonin, taurine	Several metabolic pathways differentiate ASD from TD, highlighting the role of the gut–brain axis in ASD’s pathophysiology.
**Gevi et al., 2020 [41]**	40 ASD 40 TD	To analyze the altered metabolic pathway involved in neurotransmitter production in ASD.	-	Urine	↑ homovanillic acid, 4-cresol, vitamin C (ascorbate), dopamine, glutamate ↓ MHPG, vanillylmandelic acid, pyridoxal phosphate, noradrenalin, adrenalin, homovanillic acid, GABA	The identification of several urinary metabolites involved in the dopamine pathway explains the involvement of the gut microbiota in regulating neurodevelopmental disorders (“microbiota-gut-brain axis”).
**Ma et al., 2021 [42]**	117 ASD 119 TD	To identify metabolic variations between ASD and TD.	ADOS-2 ADI-R	Urine	↑ glycine, guanidinoacetic acid, creatine, hydroxyphenylacetylglycine, phenylacetylglycine, formate ↓ 3-aminoisobutanoic acid, alanine, taurine, creatinine, hypoxanthine, N-methylnicotinamide	Urinary amino acid metabolites were significantly altered in children with ASD; the pathways in which they are involved play a key role in autism pathophysiology.
**Chung et al., 2021 [43]**	75 ASD 29 TD	To identify a metabolomic profile of ASD.	-	Blood	↓ O-phosphotyrosine	- A total of 191 features were associated with ASD. - Glutathione metabolism was affected in ASD. - exogenous chemicals (pharmaceuticals, natural dietary molecules, food additives) are significantly associated with ASD. - O-phosphotyrosine endogenous metabolite was associated with a decreased risk of ASD.
**Corraud et al., 2021 [44]**	37 ASD 37 TD	Application of newborns’ DBS for the study of metabolomic abnormalities related to ASD.	-	Blood	↑ methacholine in newborns later diagnosed with ASD	Methacholine structural analog was found at a higher—although not significant—abundance in newborns that were diagnosed with ASD by age 7, suggesting it is a relevant early marker for ASD.
**Timperio et al., 2022 [45]**	30 ASD 30 SIBS	To detect metabolic fingerprint related to ASD.	VABS	Urine	↑ thiamine-phosphate, deoxyribose-phosphate, hypoxantine, guanine, cystine, acetylysine, hypoxanthine, xanthosine, phenylalanine, p-cresol ↓ tyrosine	Phenylalanine–tyrosine–tryptophan metabolism, phenylalanine metabolism, purine metabolism and glutathione metabolism represent the most perturbed metabolic pathways in ASD.
**Khan et al., 2022 [46]**	65 ASD 20 TD	To identify potential biomarkers for ASD diagnosis.	-	Urine	↑ **Increased**: indole acetic acid, adipic acid, suberic acid, homovanillic acid, 3-hydroxy butyric acid, aconitic acid, succinic acid, citric acid, palmitic acid, lactic acid, 2-ketoglutaric acid, hippuric acid, 5-hydroxymethyl-2-furoic acid, pimelic acid ↓ **Decreased**: 3-hydroxy isovaleric acid	3-hydroxy isovaleric acid, homovanillic acid, adipic acid, suberic acid and indole acetic acid represent the most discriminant metabolites between the ASD and TD groups.

Legend: increased concentrations (↑); decreased concentrations (↓); autism spectrum disorder (ASD); siblings (SIBS); typical development (TD); developmental delay (DD); Down syndrome (DS); fragile X syndrome (FSX); fragile X mental retardation protein (FMRP); dried blood spot (DBS); autism diagnostic interview-revised (ADI-R); global assessment of functioning (GAF); (WAIS) autism diagnostic observation schedule-2nd edition (ADOS-2); autism diagnostic observation schedule-generic (ADOS-G); social communication questionnaire (SCQ); autistic behavior checklist (ABC); childhood autism rating scale (CARS); developmental quotient (DQ); intelligence quotient (IQ); Vineland Adaptive Behavior Scales (VABS).

**Table 2:** This table includes 8 studies employing metabolomic analyses to characterize the clinical ASD phenotypes in terms of core symptom severity (AIM 2).

Association between Metabolome and ASD Clinical Phenotype						
Study	Sample	Aim of the Study	Clinical Tools	Biofluid	Main Findings of Metabolities in ASD	Main Authors’ Conclusions
**Altieri et al., 2011 [47]**	59 ASD 59 TD	To measure *p*-cresol to assess possible pathophysiological roles of the gut in ASD.	ADOS-2 ADI-R CARS VABS IQ	Urine	↑ *p*-cresol	Higher levels of *p*-cresol in: - ASD children compared to TD - ASD children < 8y/o - females compared to males - individuals with history of behavioral regression
**Rangel-Huerta et a. 2019 [22]**	30 ASD 30 TD	To evaluate the metabolomic profiles of children with ASD with (AR) and without regression (ANR).	PDDBI Battelle developmental test ADI-R	Blood	**Differences between TD and ASD:** In ASD: ↑ **increase of:** 4-methyl-2-oxopetane, arginine, tryptophan, homoarginine, n-alpha-acetylornithine, N-acetylarginine, 1-methyl-nicotinamide and N-methyl-2-pyridone-5-carboxamide ↓ **decrease of:** glutamate, aspartate, nicotinamide, 1-palmitoyl-glycerol-phosphatidyl-etholamine (GPE), 1-stearoyl-GPE **Differences between AR and ANR:** *ASD-ANR*: ↑ 3-methylxanthine, 7-methylurate and 3-methylhistidine kynurenine, 5-bromotryptophan, 3-indoxyl sulfate, indolelactate, 6-hydroxyindole sulfate, *ASD-AR*: ↑ several species of mono-and diacylglycerols ↓ ilaurate, myristate and palmitate (free fatty acid)	- Several differences between TD and ASD children were detected, involving mainly amino acid, lipid and nicotinamide metabolism. - subtle differences between the ANR and AR group; - AR phenotype present alterations of amino acid, NAD^+^ and lipid metabolism, (arginine and glutamate pathway)
**Mussap et al., 2020 [21]**	31 ASD 26 TD	To explore metabolic perturbations in ASD: -to investigate the possible association between the severity of core symptoms and specific metabolic signatures; - to examine whether the urine metabolome discriminates severe from mild-to-moderate restrictive and repetitive behaviors	ADOS-2 RBS-R ABC-C	Urine	↑ **Increase of:** hypoxanthine, allantoin, lactic acid, succininc acid, quinic acid (more pronounced ASD-FS), hippuric acid (less pronounced in ASD-FS), tryptophan, indole-3-acetic acid, quinolinic acid, 5-hydroxyindoleacetic, 2-hydroxylacrylic, *p*-cresol, trihydroxypentanoic acid, HPHPA ↓ **Decrease of:** 7-methylxanthine (less pronunced in ASD-FS), uric acid, scylloinositol, kynurenic acid	The severity of ASD core symptoms and problematic behaviors may be associated with specific metabolic perturbations, most of them induced by an overgrowth of *Clostridia* spp., changes in the gut microbiome (e.g., overgrowth of *Candida* sp.), and by alterations in mitochondrial functions.
**Laghi et al.** **2021 [24]**	80 ASD (6 low ADOS-2; 42 moderate ADOS-2; 32 high ADOS-2) With or without GI symptoms	To identify possible correlations between metabolome, microbiota, calprotectin levels and ASD symptoms severity	ADOS-2 GSI	Fecal	**ASD- GI vs. ASD-NGI:** ↑ acetate, formate, orotate, propionate, uridine ↓ alanine, ethanol, isoleucine, leucine, methionine, phenylalanine, tyrosine **High-ADOS vs. Low-ADOS:** ↑ fucose, 1,3-dihydroxyacetone, N-methylhydantoin	- Close relationship between the ASD severity and the fecal metabolomic profile. - No links between ASD severity or GI symptoms and gut species relative abundance; - fecal metabolome discriminates the ASD severity and intestinal microorganisms mediate the link between metabolome and ASD severity, regardless of GI symptomatology.
**Needham et al., 2021 [48]**	130/231 ASD 101 TD	To determine molecular signature of ASD and its correlation with clinical phenotypes	ADOS ADI-R	Fecal Blood	**Correlation between metabolomic profile and ASD symptoms:** - Positive correlations: cysteine, methionine, glutathione, energy metabolites Negative correlations: gamma-glutamyl amino acids	- Differences in amino acid, lipid, and xenobiotic metabolism discriminate ASD and TD groups; - Specific metabolic profiles correlates with clinical behavior scores.
**Zhu et al., 2021 [49]**	120 ASD 60 TD	Investigate gut metabolomic profile and its interaction with clinical phenotype	ABC(a) SRS CARS Gesell Developmental Scale	Fecal	**Differences between ASD and TD:** ↑ **Increase of:** xanturenic acid, 5-Hydroxy-N-formylkyurenine, 5-Hydroxytryptophan, serotonin, N-Feruloyl serotonin, homocysteine ↓ **Decrease of:** 6-Hydroxymelatonin, 5-Hydroxy-indoleacetic acid, DHF, glycine, N-feruloyl glycine, 5-MTHF, carnitine, N-Acetyl-cisteine, S-Aminoethyl-L-cysteine, sulfurous acid, glutamine, GABA, indole-acetyl glutamic acid, N-Phenylacetyl glutamic acid, S(PGA1)-glutathione, glutathionyl spermidine, agmatine, p-coumaroyl agmatine, spermidine, spermine, N1,N12-Diacetyl spermine **Correlations between metabolomic profile and ASD symtpoms:** - Positive correlations: retinol, Hcy, serotonin, N-feruloyl serotonin, 5-HIAA - Negaative correlations: agmtine, S-aminoethyl-L-cysteine, 6-hydroxymelatonin, pyridoxamine, GABA, 5-trans prostaglandin F2β	The main discriminant gut metabolites involved in ASD are related to vitamin and amino acid pathways; Vitamin and amino acid metabolism pathways – with a stronger enrichment for tryptophan, retinol, cysteine-methionine metabolism- represents the most metabolic alteration in ASD sample and are correlated with symptoms severity.
**Wang et al., 2022 [50]**	29 ASD 30 TD	To identify plasma metabolic signature of ASD; To explore the relationship between metabolomic aberration and clinical profiles	ABC(a) CARS Gesell Developmental Schedule	Blood	**Differences between ASD and TD:** ↑ **Increase of:** l-Valine, palmitoleic acid, epsilon-caprolactam, arachidonic acid, prostaglandin D2. ↓ **Decrease of:** choline, aminoimidazole ribotide), 1-Acylglycerophosphocholine, deoxyribose, benzoic acid, 3-Butynoate, ornithine **Correlation between metabolomic profile and ASD symptoms:** *Negative correlation:* choline and ornithine	- Upregulation of arachidonic acid metabolism differentiate the ASD group from the TD group; - Ornithin may represent a potential discriminant biomarker in ASD; - Ornithin and Choline levels are related with aberrant behaviors.
**Brister et al., 2022 [51]**	57 ASD 37 TD	To target metabolic signature related to ASD; To evaluate the link between metabolomic aberration and clinical profile (behavior and cognition) among ASD.	VABS ABC SRS CELF PLS-5 CSHQ	Blood	**Differences between ASD and TD:** ↑ **Increase of:** cytidine, taurine, glycine, 1-methylhistamine, adenosine triphosphate, 2,3,4,5-tetrahydroxypentanoic acid, dihydroxyacetone, 4-pyridoxic acid, Xylitol, Isobutyric acid, L-Histidine, DL-Acetylcarnitine, pyroglutamic acid ↓ **Decrease of:** 5-Aminolevulinic acid, dodecanoic acid, 4-hydroxyproline, phenylpyruvic acid, capric acid, D-Leucic acid, L-2 Phenyllactic acid, 4-Hydroxybenzaldehyde **Differences between ASD with and without NDR** with NDR: ↑ niacinamide, acetamide without NDR: ↓ 2-Pyrocatechuic acid	- 23 metabolites related to Histidine metabolism, GSH metabolism and AAA biosynthesis were found to be significant different between ASD and TD; - alteration in nicotinamide metabolism differentiated the most the ASD group from the controls.

Legend: increased concentrations (↑); decreased concentrations (↓); autism spectrum disorder (ASD); typical development (TD), autism diagnostic observation schedule-2nd edition ADOS-2), gastrointestinal (GI), autistic with regression (AN); autistic without regression (ANR), autistic with food selectivity (ASD-FS); autism diagnostic interview-revised (ADI-R); childhood autism rating scale (CARS); vineland adaptive behavior scales (VABS); pervasive developmental disorders behavior inventory (PDDBI); repetitive behavior scale-revised (RBS-R); aberrant behavior checklist-community (ABC-C); autism behavior checklist (ABC(a)); gastrointestinal severity index (GSI), autism behavior checklist (ABC), social responsiveness scale (SRS), clinical evaluation of language fundamentals (CELF), preschool language scale-5 (PLS-5), childhood sleep habits questionnaire (CSHQ), neurodevelopmental regression (NDR).

**Table 3:** This table includes 7 studies investigating the possible use of metabolomics to identify co-occurring medical conditions among the ASD clinical population (AIM 3).

Associations between ASD, Metabolomes and Medical Comorbidities						
Study	Sample	Aim of the Study	Clinical Tools	Biofluid	Main Findings of Metabolites in ASD	Main Authors’ Conclusions
**Gevi et al.** **2016 [25]**	30 ASD 30 TD	To detect differences in urinary metabolic patterns.	-	Urine	↑ **Increased:** xanthurenic acid, quinolinic acid, indoxyl sulfate, indole derivatives (indolyl-3-acetic acid, and indolyl lactate), inosine, hypoxanthine, xanthosine, uridine, phenylalaine, 6-phospho-d-gluconic acid, thiamine, tryptophan, riboflavin, p-cresol, 4-pyrodic acid, histidine, trehalose/sucrose, xanthosine ↓ **Decreased:** kynurenic acid, melatonin, n-acetyl-5-methoxy- tryptamine cellobiose, pyroglutamic acid, methionine, p-hydroxybenzoate, pantothenate, adp, ribose, glucose-6-phosphate, indole-3-carboxylic acid	Perturbed metabolic pathways in ASD: - Tryptophan metabolism - Purine metabolism - Vitamin B6 metabolism - Phenylalanine–tyrosine–tryptophan biosynthesis The tryptophan metabolic pathway collectively displays the largest perturbations in ASD. These metabolic abnormalities could underline several comorbidities frequently associated with ASD, such as sleep disorders and gastrointestinal symptoms, and could contribute to autism severity.
**Gabriele et al., 2016 [52]**	53 ASD	To investigate potential factors contributing to elevated urinary p-cresol levels in ASD.	GMDS	Urine Fecal	↑ p-cresol (the total p-cresol measured here is actually the sum of free p-cresol and its two conjugated derivatives, namely p-cresylsulfate and p-cresylglucuronate)	The results support a primary role for slow intestinal transit in linking ASD to elevated urinary p-cresol, which seemingly characterizes most autistic children with chronic constipation.
**Kang et al., 2018 [53]**	21 ASD 23 TD	To detect fecal metabolites’ differences between ASD and TD and to further investigate the link with the gut microbiome.	-	Fecal	↑ isopropanol, p-cresol ↓ GABA	The results show discriminant fecal metabolomic profiles between ASD and TD and confirm the reduced abundances of microbial species related to *Prevotella copri* in the ASD clinical group.
**Hua et al., 2020 [54]**	120 ASD (60 with and 60 without SDs)	- To investigate changes in gut microbiota. – To evaluate changes in metabolites. – To evaluate their correlation with ASD’s core symptoms and sleep problems.	ABC SRS CSHQ	Fecal	**In ASD+SDs**: ↑ serotonin ↓ 3-hydroxybutyric acid, melatonin	The results suggest that there were changes in fecal metabolites related to key gut microbiota components in children with ASD suffering from sleep problems. The 3-hydroxybutyric acid level may influence the melatonin level, which is associated with sleep disorders.
**Dan et al., 2020 [55]**	143 ASD 143 TD (30 C-ASD; 30 NC-ASD; 30 TD)	- To understand the relationship between gut microbiota and fecal metabolites in ASD. - To investigate the potential interaction between ASD and GI symptoms.	-	Fecal	↑ **Increased:** quinic acid, 3-Dehydroquinate, thr-Phe, desaminotyrosine, vanillactic acid, indole-3-carboxylic acid, hexanoic acid, 3-Indoxyl-D-Glucopyranoside, 2,5-dioxopentanoate, γ-glutamylglycine, phosphatidylcholine, D-4′-phosphopantothenate, pantothenic acid, 3-(uracil-1-yl)-L-alanine, 3-Dehydrocarnitine, methylselenocysteine selenoxide, deoxyinosine, 1-methyladenosine, orotidine-5P 2′-deoxyuridine ↓ **Decreased:** tyr-leu, DL-P-Hydroxyphenyl lactic acid, indoleacetaldehyde, imidazole-4-acetaldehyde, adenine, deoxyadenosine, 2′-deoxyguanosine	The interaction analysis between gut microbiota, metabolites and neurotransmitters provides clues for better understanding the mechanisms underlying altered social behaviors in ASD.
**Daneberga et al., 2022 [56]**	44 ASD	To identify urinary metabolites as biomarkers for GI disorders.	-	Urine Microbiome composition	No significant differences in quantified metabolites compared with reference values. **Correlations between metabolomic and microbiome profiles**: ↑ p-cresol and ↑ *Firmicutes phylum* ↑ HPHPA and ↓ *Bacteroidetes/Firmicutes ratio*	Urinary metabolites can be used as biomarkers for GI alterations with caution and not solely. A combination of urinary organic acid and microbiota analyses is necessary to assess a treatment for gastrointestinal issues.
**Kang et al., 2020 [57]**	18 ASD 20 TD	Determine which metabolites were different in the ASD group before and after MTT.	-	Fecal Plasma	**At the baseline in ASD group:** ↑ caprylate, heptanoate ↓ nicotinamide, riboside, IMP, iminodiacetate, methylsuccinate, galactonate, valyglycine, sarcosine, lecyglycine	Children with ASD present different levels of plasma metabolites at baseline compared with TD. MTT promoted changes in plasma metabolites, driving a number of metabolites to be more similar to those from TD children.

Legend: increased concentrations (↑); decreased concentrations (↓); autism spectrum disorder (ASD); typical development (TD); sleep disorders (SDs); autistic with chronic constipation (C-ASD); autistic without chronic constipation (NC-ASD); gastrointestinal (GI); microbiota transfer therapy (MTT); Griffiths mental development scale (GMDS); aberrant behavior checklist (ABC); social responsiveness scale (SRS); children’s sleep habits questionnaire (CSHQ).

**Table 4:** This table summarizes the main metabolites discussed in the overview. Detailed description of type of metabolites, pathway in which they are involved, mechanisms of production and their respective physiological and pathological functions are reported (source: the Human Metabolome Database—HMDB https://hmdb.ca, accessed on the 26 January 2023).

	P-Cresol	2-Hydroxyacrylic Acid	3-Hydroxy Pentanoic Acid	HPHPA	3-Hydroxy Butyric Acid	Kynurenic, Quinolinic and Xanthurenic Acids	Serotonin	Melatonin
**Metabolite**	Organic aromatic compound	Carboxylic acid, organic	Short-chain fatty acid (pentanoic acid)	Abnormal phenylalanine metabolite	Straight-chain 3- hydroxy monocarboxylic	Urinary metabolites	Indoleamines	Biogenic amine
**Pathway**	Tyrosine biosynthesis	Not available	Not available	Flavan-3-ol	- Fatty acid biosynthesis - Ketone body metabolism	Tryptophan	Tryptophan	Tryptophan
**Production**	Intestinal microflora, fecal biomarker of Clostridium difficile infection	Intestinal microflora; produced by *Clostridia* spp. through the reduction of lactic acid to propionic acid via an acrylyl-CoA intermediate	Liver Commonly found in human feces and produced by different *Clostridia* spp. and gut bacteria	GI tract, caused by *Clostridia* spp.	Released from muscles for hepatic and renal gluconeogenesis	- Peripheral cells and tissues, GI microbiota (kinurenic acid) - Brain (quinolinic and xanthurenic acids)	GI tract, CNS and blood from the amino acid L-Tryptophan	Brain (pineal gland)
**Physiological** **function**	Antimutagenic, antiseptic, cancer-preventive	Not available	Neuroprotective agent	Waste product	Involved in melatonin production; energy source for extrahepatic tissues (brain, lung)	Oxydoreduction processes that influence neural development	Biochemical messenger involved in sleep, arousal state and sexual behavior	Regulator of circadian rhythms, mood, learning, memory, immune activity and reproduction
**Pathological function**	Abnormal concentrations cause fatigue, uremia and hypoglycemia	Not available	Abnormal concentrations cause propionic acidemia	Increased levels are neurotoxic and metabotoxic	Interacts with butyrate metabolism that directly influences the release of serotonin via the vagus nerve, leading to a reduction in melatonin secretion and arising sleep problems	Role in the oxidative stress balance (decreases in kynurenine and kynurenic acid; increases in quinolinic acid and xanthurenic acid)	Its deficiency causes sleep disorders; impact on psychiatric symptoms (depression, OCD, phobias, PTSD, anxiety)	Altered concentrations interact with the circadian organization of physiological functions (sleep cycle, immune activity, antioxidative status, glucose regulation)

## Data Availability

Data sharing not applicable.
